# Supplementary material for: Time-series transcriptome analysis identified differentially expressed genes in broiler chicken infected with mixed Eimeria species
Source: Front Genet. 2022 Aug 8;13:886781. doi: 10.3389/fgene.2022.886781 (PMC9393255; doi:10.3389/fgene.2022.886781)
Supplement: Supplementary file 2 [file DataSheet1.ZIP › 7dpi_KEGG.Gsea.1625115961950/KEGG_GLIOMA.html]

Details for gene set KEGG\_GLIOMA[GSEA]

|  || Dataset | TMM\_7dpi\_gct\_format\_7dpi\_gct\_format.Class\_4dpi.cls #PC\_versus\_NC.Class\_4dpi.cls #PC\_versus\_NC\_repos |
| Phenotype | Class\_4dpi.cls#PC\_versus\_NC\_repos |
| Upregulated in class | 0 |
| GeneSet | KEGG\_GLIOMA |
| Enrichment Score (ES) | -0.48552424 |
| Normalized Enrichment Score (NES) | -1.6015897 |
| Nominal p-value | 0.0055840965 |
| FDR q-value | 0.080626905 |
| FWER p-Value | 0.676 |
Table: GSEA Results Summary

  

Fig 1: Enrichment plot: KEGG\_GLIOMA      
 Profile of the Running ES Score & Positions of GeneSet Members on the Rank Ordered List

  

| SYMBOL | TITLE | RANK IN GENE LIST | RANK METRIC SCORE | RUNNING ES | CORE ENRICHMENT || 1 | CDKN1A | na | 843 | 0.575 | -0.0501 | No |
| 2 | HRAS | na | 1342 | 0.420 | -0.0769 | No |
| 3 | TGFA | na | 1901 | 0.287 | -0.1133 | No |
| 4 | SHC4 | na | 2866 | 0.143 | -0.1889 | No |
| 5 | MAP2K2 | na | 3435 | 0.070 | -0.2339 | No |
| 6 | CAMK2B | na | 3958 | 0.009 | -0.2772 | No |
| 7 | PDGFB | na | 4266 | -0.026 | -0.3019 | No |
| 8 | PDGFA | na | 4308 | -0.031 | -0.3043 | No |
| 9 | RAF1 | na | 5142 | -0.116 | -0.3698 | No |
| 10 | MAP2K1 | na | 5188 | -0.120 | -0.3693 | No |
| 11 | SHC2 | na | 5501 | -0.149 | -0.3901 | No |
| 12 | SHC1 | na | 5796 | -0.179 | -0.4083 | No |
| 13 | PLCG1 | na | 6241 | -0.228 | -0.4374 | No |
| 14 | GRB2 | na | 6686 | -0.274 | -0.4648 | No |
| 15 | PDGFRA | na | 6935 | -0.300 | -0.4749 | Yes |
| 16 | AKT3 | na | 7033 | -0.312 | -0.4719 | Yes |
| 17 | E2F1 | na | 7190 | -0.328 | -0.4733 | Yes |
| 18 | KRAS | na | 7206 | -0.330 | -0.4629 | Yes |
| 19 | CALM2 | na | 7385 | -0.350 | -0.4654 | Yes |
| 20 | NRAS | na | 7474 | -0.358 | -0.4600 | Yes |
| 21 | CDK6 | na | 7686 | -0.382 | -0.4641 | Yes |
| 22 | CAMK2G | na | 7730 | -0.388 | -0.4540 | Yes |
| 23 | CALM1 | na | 8103 | -0.435 | -0.4696 | Yes |
| 24 | RB1 | na | 8176 | -0.443 | -0.4600 | Yes |
| 25 | AKT1 | na | 8399 | -0.472 | -0.4618 | Yes |
| 26 | PIK3CG | na | 8478 | -0.481 | -0.4513 | Yes |
| 27 | PTEN | na | 8620 | -0.503 | -0.4453 | Yes |
| 28 | PRKCA | na | 8704 | -0.513 | -0.4340 | Yes |
| 29 | PDGFRB | na | 8726 | -0.517 | -0.4174 | Yes |
| 30 | SOS1 | na | 8869 | -0.533 | -0.4104 | Yes |
| 31 | PIK3R2 | na | 9597 | -0.645 | -0.4484 | Yes |
| 32 | IGF1R | na | 9854 | -0.683 | -0.4456 | Yes |
| 33 | PRKCB | na | 9867 | -0.685 | -0.4223 | Yes |
| 34 | E2F3 | na | 10031 | -0.717 | -0.4105 | Yes |
| 35 | MTOR | na | 10269 | -0.763 | -0.4033 | Yes |
| 36 | MAPK3 | na | 10320 | -0.774 | -0.3800 | Yes |
| 37 | PIK3CB | na | 10335 | -0.777 | -0.3537 | Yes |
| 38 | CCND1 | na | 10432 | -0.796 | -0.3335 | Yes |
| 39 | PLCG2 | na | 10697 | -0.853 | -0.3253 | Yes |
| 40 | PIK3CD | na | 10707 | -0.855 | -0.2958 | Yes |
| 41 | CAMK2D | na | 10708 | -0.856 | -0.2654 | Yes |
| 42 | MDM2 | na | 10838 | -0.889 | -0.2447 | Yes |
| 43 | PIK3CA | na | 10920 | -0.912 | -0.2192 | Yes |
| 44 | PIK3R5 | na | 10941 | -0.918 | -0.1883 | Yes |
| 45 | PIK3R3 | na | 11045 | -0.949 | -0.1633 | Yes |
| 46 | CDKN2A | na | 11228 | -1.009 | -0.1427 | Yes |
| 47 | PIK3R1 | na | 11265 | -1.023 | -0.1095 | Yes |
| 48 | SOS2 | na | 11362 | -1.063 | -0.0798 | Yes |
| 49 | EGFR | na | 11415 | -1.085 | -0.0457 | Yes |
| 50 | BRAF | na | 11724 | -1.264 | -0.0267 | Yes |
| 51 | IGF1 | na | 11859 | -1.426 | 0.0126 | Yes |
Table: GSEA details [plain text format]

  

Fig 2: KEGG\_GLIOMA      
 Blue-Pink O' Gram in the Space of the Analyzed GeneSet

  

Fig 3: KEGG\_GLIOMA: Random ES distribution      
 Gene set null distribution of ES for **KEGG\_GLIOMA**
